# Primary spontaneous pneumothorax

**DOI:** 10.4322/acr.2023.468

**Published:** 2024-01-25

**Authors:** Yago Marcos Pessoa-Gonçalves, Ana Clara Vaz e Silva, Carlo José Freire Oliveira, Sheila Jorge Adad, Lucinda Calheiros Guimarães

**Affiliations:** 1 Universidade Federal do Triângulo Mineiro (UFTM), Laboratório de Imunologia e Bioinformática, Uberaba, MG, Brasil; 2 Universidade Federal do Triângulo Mineiro (UFTM), Unidade de Análises Clínicas e Anatomia Patológica, Uberaba, MG, Brasil

**Keywords:** Pleura, Pleurodesis, Pneumothorax

Pneumothorax is the presence of air in the pleural space, clinically classified as spontaneous when no identifiable precipitating factors exist and non-spontaneous when such factors are present. Further subdivision delineates into primary in the absence of an evident clinical cause and secondary when a causal relationship exists with an underlying medical condition. Thus, primary spontaneous pneumothorax (PSP) is characterized by air in the pleural space in patients without manifest clinical or pulmonary disorders.^1,2^

The annual incidence of PSP ranges from 7.4 to 18 cases per 100,000 in males and 1.2 to 6 cases per 100,000 in females.^3^ Patients with PSP typically exhibit a phenotype characterized by leanness and tall stature. Predisposing factors such as family history, Marfan syndrome, thoracic endometriosis, smoking, bronchiolitis, Birt-Hogg-Dubé syndrome, and homocystinuria may be associated with this entity. Changes in atmospheric pressure can also precipitate PSP.^3,4^ It commonly occurs at rest and is not correlated with physical activity. Most patients with PSP experience sudden ipsilateral pleuritic pain. Chest radiography and clinical history provide a highly accurate diagnosis, while occasional cases with small air volumes may require a computed tomography for diagnosis.^1,4^

Many authors^5-7^ suggest that spontaneous rupture of a subpleural bleb or bulla causes PSP. However, the mechanism behind the accumulation of subpleural air has yet to be clearly described. Currently, the likely cause is believed to be the degradation of lung elastic fibers induced by factors like smoking. This degradation increases neutrophils’ and macrophages’ concentration, thereby enhancing elastic degradation.^1,3^ Air can enter the interlobular septum through alveolar ruptures and accumulate in the subpleural regions. Additionally, pleural porosity has been described as an area where disruption of mesothelial cells in the visceral pleura occurs, leading to their replacement by fibroblastic tissue and increased porosity that permits air to enter the subpleural space.^5,6^

The treatment of PSP remains controversial, lacking a definitive consensus to date. Some studies indicate that outpatient treatment or sole pleural drainage results in a 38% recurrence rate of PSP. Conversely, patients treated with bullectomy, involving the surgical removal of blebs typically situated at the apex of the lung, combined with pleurodesis—a procedure inducing a fibrotic reaction in the pleura—demonstrate no recurrence.^8,9^ Ongoing debate surrounds the necessity of routine anatomopathological analysis post-surgery for all patients with PSP, as it might not change the prognosis in numerous cases.^5-7^

In this report, we present the gross and histopathological findings from a 20-year-old male patient. He stood at 192 cm, weighed 75 kg, with a body mass index of 20.35, and experienced sudden left thoracic pain and dyspnea while walking. An imaging workup revealed a significant air volume in the pleural cavity along with numerous apical blebs in the left lung. Subsequently, the treatment involved apical segment excision of the left lung, followed by pleurodesis.

**Figure 1:**
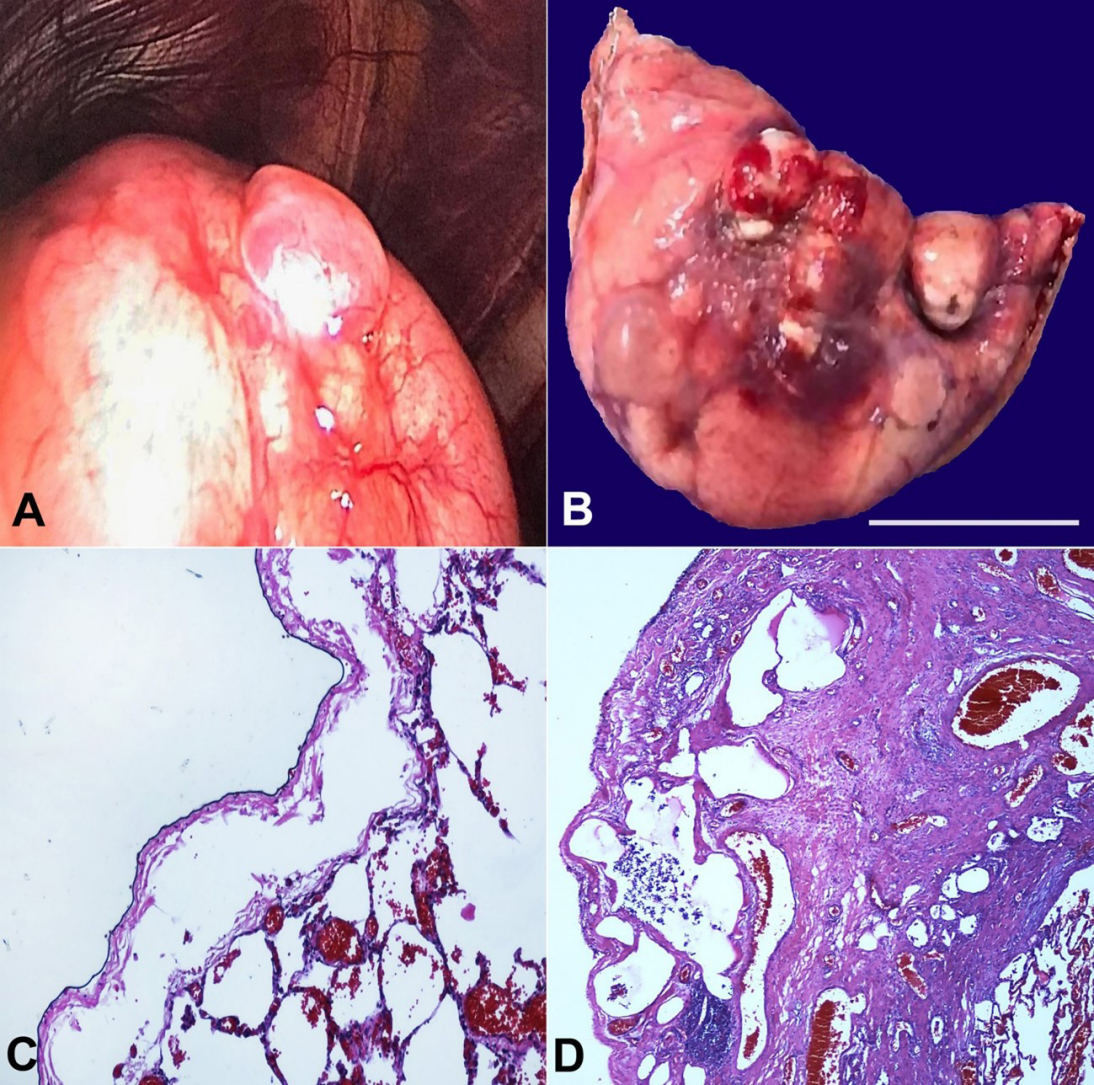
**A -** video-assisted thoracoscopy reveals a bulla surrounded inferiorly by an area with increased vascularity and pleural retraction; **B -** surgical specimen from the lung apex with several bullae (scale bar= 2cm); **C -** areas exhibiting subpleural and intrapleural air accumulation (H&E; 100x); **D -** pleural porosity characterized by intense fibrous thickening of the visceral pleura, interspersed with air within the fibrotic regions (H&E; 100x).

During the surgical approach using video-assisted thoracoscopy, we observed a sizable bulla surrounded by an area displaying increased vascularity and pleural retraction inferiorly, likely attributed to the prior rupture of another bulla or inflation (Figure 1A). The surgical specimen obtained from the apex of the left lung showed several bullae containing hematic contents, likely due to surgical manipulation, alongside areas consistent with previously ruptured bullae (Figure 1B). Microscopic analysis revealed areas demonstrating subpleural and intrapleural air accumulation, such as pleural interstitial emphysema (Figure 1C). Evidence of pleural porosity, characterized by intense fibrous thickening of the visceral pleura interspersed with air within the fibrotic regions, was observed (Figure 1D). The microscopic findings are significant as they correspond with the presumed mechanism involved in bleb formation.
